# Circulating tumor DNA moves further into the spotlight

**DOI:** 10.1186/gm552

**Published:** 2014-05-28

**Authors:** Mark Sausen, Sonya Parpart, Luis A Diaz

**Affiliations:** 1 Personal Genome Diagnostics, Baltimore, MD 21224, USA; 2 Ludwig Center for Cancer Genetics and Therapeutics, Howard Hughes Medical Institute and the Sidney Kimmel Comprehensive Cancer Center at Johns Hopkins, Baltimore, MD 21287, USA; 3 Swim Across America Laboratory at Johns Hopkins, Baltimore, MD 21287, USA

## Abstract

Assessment of somatic genomic alterations from tumors can now be performed by sequencing circulating tumor DNA from the cell-free component of blood. This procedure, which identifies tumor-derived somatic mutations from a simple blood sample, circumvents the need for tumor tissue. A recent study highlights the promise of circulating tumor DNA to guide therapeutic decisions in a variety of solid tumors for both clinical and investigative purposes, as well as providing a tool for the early detection of cancer.

## 

The presence of cell-free DNA in the circulation was first formally described by Mandel and Metais in 1948 [1]. Soon after, circulating tumor DNA (ctDNA) was noted to be the tumor-derived fraction in the cell-free DNA component of blood [2]. ctDNA can be detected across many common solid tumor types in patients in both early and late stages of cancer, with levels ranging from less than one to greater than 100,000 mutant DNA fragments per milliliter of plasma [3-5]. The utility of ctDNA as a cancer biomarker hinges on the exquisite specificity of somatic genomic alterations in patients with cancer. Unlike other tumor biomarkers, the specificity of somatic alterations is derived from the fact that mutations are present in the genome(s) of tumor cells but not in the genome of matched normal cells.

Many advances in this area have been possible through recent developments in digital genomic approaches (for example, digital PCR). These methods allow for the identification of somatic, or tumor-specific, mutations with allele fractions as low as 0.01% in a wild-type background [5]. Advances in digital genomics have opened the door for reliably evaluating very rare events in complex mixtures of tumor-derived and wild-type DNA. As a result, there has been a flurry of studies that detail the biology and clinical applicability of ctDNA [3,6,7].

One such recent study by Newman *et al*. [8] describes a novel cancer profiling method for non-small cell lung cancer (NSCLC) using deep sequencing (CAPP-Seq; cancer personalized profiling by deep sequencing) to quantify ctDNA. This approach allows the evaluation of specific regions of interest contained within ctDNA, and results in a significant cost reduction and improved sensitivity over whole-exome- and whole-genome-based approaches. A targeted 139 gene panel, or ‘selector’, for NSCLC was designed by analyzing whole-genome sequencing data for several types of somatic alterations, including sequence mutations and translocations, and was shown to detect >95% of cases. When applied to plasma collected from five healthy controls and 13 patients with NSCLC, CAPP-Seq achieved a sensitivity of 85% and specificity of 96% for detection of ctDNA. A sensitivity of 50% was achieved for patients at stage I and 100% for those at stages II to IV, while specificity was 96% in both groups, similar to previous evaluations of patients with early- and late-stage disease [3].

Furthermore, until now, it has been difficult to evaluate mutational heterogeneity within and among tumors in a single patient because of the need for multiple tumor biopsies. Mutational heterogeneity can also be increased after therapy [6,7]. The less invasive nature of liquid biopsies, in addition to the comprehensive information they achieve, makes this form of biopsy ideal to monitor a patient’s response to therapy and find additional mutations that may occur over time. The Newman *et al*. study highlights the utility of liquid biopsies for this purpose when examining two recurrently mutated genes, *EGFR* and *KRAS*. Their CAPP-Seq method correctly identified all mutations with allelic fractions >0.10% and yielded a specificity of 99%. Somatic mutations included two different *EGFR* mutations: an activating mutation in a dominant tumor clone as well as a second T790M mutation present in an erlotinib-resistant subclone. Of note, the mutations were identified in both the tumor tissue and ctDNA of this patient. Though evidence of multiple clones was found in only a single patient, this example demonstrates the ability of ctDNA to both address tumor heterogeneity and find clinically relevant mutations stemming from subclonal tumor populations.

The work presented by Newman and colleagues is yet another step forward as it advances the practicality of using ctDNA. However, the next phase of studies in the field of ctDNA will be critical to define the clinical value of this technology. What are the key next steps? First, we need to better understand biologic sensitivity; in other words, how much ctDNA is present in the circulation at each stage of disease and across disease types. Initial studies have begun to probe this question but, as larger clinical studies are contemplated, it is critical to understand both the variability in the amounts of ctDNA present and the etiology of this variability.

Second, we need to move beyond point mutations in order to appreciate the full spectrum of genomic alterations that include aneuploidy, amplifications, deletions, and translocations, especially because these represent some of the most clinically useful genomic alterations in cancer (for example, *ERBB2* amplifications). This work has begun, but only in small pilot studies, with larger studies needed to further define these structural alterations in ctDNA [9,10].

Finally, definitive clinical studies that make use of ctDNA are crucial to advance liquid biopsy tests into widespread practice. This will require standardization of a platform of choice, defining the technical variability of this platform, and then performing a properly designed prospective study that addresses a key clinical question involving ctDNA.

Studies like the one by Newman *et al*. [8] and others [3] continue to bring ctDNA into the spotlight and highlight the various areas in which it shows clinical potential. The next phase of studies will further illuminate the path we need to take to bring ctDNA into the clinic as a meaningful biomarker - one that has the potential to address some of the toughest clinical problems in oncology.

## Abbreviations

CAPP-Seq: Cancer personalized profiling by deep sequencing; ctDNA: Circulating tumor DNA; NSCLC: Non-small cell lung cancer.

## Competing interests

LAD owns Personal Genome Diagnostics stock, which is subject to certain restrictions under University policy. The terms of these arrangements are managed by the Johns Hopkins University in accordance with its conflict-of-interest policies. The other authors declare that they have no competing interests.
